# EXPLORING THE HEALTH BURDEN AND SOCIOECONOMIC COSTS OF DEPENDENT ELDERLY CARE IN NSUKKA, NIGERIA

**DOI:** 10.1093/geroni/igz038.490

**Published:** 2019-11-08

**Authors:** Peter C Ezeah

**Affiliations:** NNAMDI AZIKIWE UNIVERSITY, AWKA, ANAMBRA, Nigeria

## Abstract

It is projected that by 2030, 6 percent of Nigeria’s present population of 180 million will be 60 years and above. However, the extent to which the traditional systems of family support and security can manage the care of the increasing number of older people in the country is not clear as limited studies are available in the country regarding the health burden and Socioeconomic costs of caring for dependent older people. This study is therefore aimed at assessing the health burden and costs of caring for dependent older people in Nsukka, Nigeria. This cross sectional survey involved 1030 randomly selected elderly persons in Nsukka, Nigeria (Mage=70.15, SD=12.23). Structured questionnaire and Focus Group Discussion Guide (FGD) provided the data for the study. The qualitative data were analyzed with descriptive statistics, while regression analysis formed the basis for predicting effects of the variables in the study. The qualitative data from the FGD were analyzed thematically. The findings show that the Nigeria government was largely uninvolved in the care and support for older dependent people; leaving families to negotiate a ‘journey without maps’. Families carried the health burden of care for the elderly with attendant socioeconomic costs. The traditional role of female relatives as caregivers was beginning to give way to paid caregivers. An innovative policy frame work targeted at the needs of older persons in health care, social protection and other forms of intergenerational support is required to supplement inputs from families of the aged in Nigeria.

